# Molecular characterization of *Paenibacillus antarcticus* IPAC21, a bioemulsifier producer isolated from Antarctic soil

**DOI:** 10.3389/fmicb.2023.1142582

**Published:** 2023-03-21

**Authors:** Ericka Arregue de Lemos, Luciano Procópio, Fabio Faria da Mota, Diogo Jurelevicius, Alexandre Soares Rosado, Lucy Seldin

**Affiliations:** ^1^Instituto de Microbiologia Paulo de Góes, Universidade Federal do Rio de Janeiro, Rio de Janeiro, Brazil; ^2^Instituto Oswaldo Cruz, Rio de Janeiro, Brazil; ^3^Biological and Environmental Sciences and Engineering (BESE), King Abdullah University of Science and Technology, Thuwal, Saudi Arabia

**Keywords:** *Paenibacillus antarcticus*, genome, bioemulsifier, levan, Antarctica

## Abstract

*Paenibacillus antarcticus* IPAC21, an endospore-forming and bioemulsifier-producing strain, was isolated from King George Island, Antarctica. As psychrotolerant/psychrophilic bacteria can be considered promising sources for novel products such as bioactive compounds and other industrially relevant substances/compounds, the IPAC21 genome was sequenced using Illumina Hi-seq, and a search for genes related to the production of bioemulsifiers and other metabolic pathways was performed. The IPAC21 strain has a genome of 5,505,124 bp and a G + C content of 40.5%. Genes related to the biosynthesis of exopolysaccharides, such as the gene that encodes the extracellular enzyme levansucrase responsible for the synthesis of levan, the 2,3-butanediol pathway, PTS sugar transporters, cold-shock proteins, and chaperones were found in its genome. IPAC21 cell-free supernatants obtained after cell growth in trypticase soy broth at different temperatures were evaluated for bioemulsifier production by the emulsification index (EI) using hexadecane, kerosene and diesel. EI values higher than 50% were obtained using the three oil derivatives when IPAC21 was grown at 28°C. The bioemulsifier produced by *P. antarcticus* IPAC21 was stable at different NaCl concentrations, low temperatures and pH values, suggesting its potential use in lower and moderate temperature processes in the petroleum industry.

## Introduction

The genus *Paenibacillus* was created by Ash et al. (1993) to accommodate the former ‘group 3’ of the genus *Bacillus*. Currently, the genus comprises more than 280 validated species^1^ that harbor endospore-forming, low G + C gram-positive bacterial strains (Lal and Tabacchioni, 2009; Grady et al., 2016; Jeong et al., 2019). Strains belonging to the genus *Paenibacillus* have already been isolated worldwide from different types of soils and plants, deserts, caves, cold regions, and aquatic environments, many of which are of great importance to humans, animals, plants, the environment and/or as biotechnological agents in industrial processes (Lal and Tabacchioni, 2009; Grady et al., 2016; Patowary and Deka, 2020).

Considering the petroleum industry, *Paenibacillus* strains are efficient in bioremediating different environments contaminated with pollutants derived from extracting, refining, and transporting petroleum and coal tar (Grady et al., 2016). Some examples are *P. ehimensis* BS1, which is able to degrade heavy crude oil (Shibulal et al., 2017), *Paenibacillus* sp. strains D2, D9 and D10 are considered diesel fuel degraders (Ganesh and Lin, 2009), and *P. naphthalenovorans* PR-N1 (Daane et al., 2002) and *Paenibacillus* sp. PHE-3 (Zhu et al., 2016), both of which are able to degrade polycyclic aromatic hydrocarbons (naphthalene and phenanthrene, respectively). Moreover, biosurfactants (or other bacterial byproducts such as bioemulsifiers, acids and solvents) produced by *Paenibacillus* sp. can be used for the bioremediation of oil (and derivatives)-contaminated soils and marine environments. The biosurfactant produced by *Paenibacillus* sp. D9 was used for the bioremediation of diesel and motor oil (Jimoh and Lin, 2020), and that from *P. dendritiformis* CN5 was used for polycyclic aromatic hydrocarbon (PAH) and motor oil sludge removal from contaminated soil and sand media (Bezza and Chirwa, 2015). *Paenibacillus* sp. #510 isolated from crude oil was able to produce a bioemulsifier with high potential to stabilize emulsions with different hydrocarbons (Gudina et al., 2015). In addition, the potential of *P. ehimensis* BS1 in microbial enhanced oil recovery (MEOR) technology has been demonstrated by the biotransformation of heavy to lighter crude oil under aerobic and reservoir conditions (Shibulal et al., 2017).

*Paenibacillus antarcticus* has been described as a psychrotolerant organism isolated from Antarctic sediment (Montes et al., 2004). The type strain of the species – 20CM^T^ – produces oxidase, catalase and urease, and it hydrolyzes aesculin, starch, and Tween 80. Psychrotolerant/psychrophilic bacteria can be considered promising sources for novel products such as bioactive compounds and other industrially relevant substances/compounds (Dhakar and Pandey, 2020). Moreover, cold-active enzymes found in psychrotolerant/psychrophilic bacteria are very interesting for different processes in industry (including the petroleum industry), pharmaceuticals, medicine, and food (Yadav et al., 2019).

Vollú et al. (2014) isolated and characterized different endospore-forming and cold-adapted bacteria from soil samples of King George Island, which is part of the South Shetlands archipelago in Maritime Antarctica. The ability to produce extracellular enzymes and antimicrobial substances and to emulsify n-hexadecane was determined in all isolates in an attempt to contribute to a potential source of cold active bioproducts for industrial use. One strain identified as *P. antarcticus* and denoted IPAC21 was able to efficiently emulsify n-hexadecane in laboratory conditions. However, no further tests have been performed to determine the surfactant effect of reducing surface tension. Although biosurfactants and/or bioemulsifiers have been extensively used in the remediation of oil-contaminated water and soil (Silva et al., 2014; Maia et al., 2019), very little is known concerning their biotechnological potential in this *Paenibacillus* species.

Therefore, we report the genomic characterization of the psychrotolerant strain IPAC21, which was isolated from soil collected in Ipanema, King George Island, Antarctica, highlighting important metabolic pathways such as those for its adaptation to environmental stress conditions and for bioemulsifier production. Moreover, the physical and chemical conditions for its production and its ability to emulsify oils derived from the petroleum industry were evaluated. Our results provide important information about the potential use of the bioemulsifier produced by *P. antarcticus* IPAC21 for future biotechnological applications.

## Materials and methods

### Bacterial strain

The bacterial strain IPAC21 was previously isolated from soil samples from Ipanema (UTM coordinates – latitude/longitude = E:426.570/N:3.116.513), King George Island, Antarctica (Vollú et al., 2014). The strain IPAC21 was stored in tryptic soy broth (TSB) containing 20% glycerol at −80°C. To evaluate the growth of IPAC21 at different temperatures, the strain was grown in TSB at 5°C, 15°C and 28°C for up to 96 h under agitation at 130 rpm.

### DNA extraction, genome sequencing and assembly

For DNA extraction, strain IPAC21 was initially cultivated in 5 ml of TSB at 28°C under agitation at 130 rpm for 48 h. Then, the culture was centrifuged at 12,000 g, the supernatant was discarded, and the pellet was recovered. DNA from the IPAC21 strain was extracted and purified according to Seldin et al. (1998) and Seldin and Dubnau (1985), respectively. Subsequently, the DNA was quantified spectrophotometrically using a Qubit™ fluorimeter (Thermo Fisher Scientific, MA, USA).

The genome of strain IPAC21 was sequenced on the Illumina HiSeq 2,500 platform as recommended by the manufacturer. Approximately 5 μg/μg DNA was used for the construction of paired-end sequencing libraries (2 × 150 bp) with a 450 bp insert size. The quality analysis of the final libraries was performed using a 2100 bioanalyzer (Agilent Technologies, CA, USA). Genomic contigs were *de novo* assembled using SPAdes software, version 3.15.5 (Prjibelski et al., 2020).

### Genome annotation

The automatic annotations of the IPAC21 genome were performed using the online server RAST (Aziz et al., 2008) and NCBI Prokaryotic Genome Annotation Pipeline (PGAP, version 2022-04-14.build6021). The KEGG^2^ and Metacyc^3^ databases were used for manual annotation and construction of metabolic pathways. The pathways according to the genome annotation of the IPAC21 strain were created with BioRender.com.

### Phylogenetic analyses

For the phylogenetic analyses, 16S rRNA-encoding gene sequences of closely related species to *Paenibacillus antarcticus* were retrieved from the GenBank database. Sequence alignments were performed using the ClustalW method in MEGA-X software. The sequences were aligned through the ClustalW method to analyze the similarity between those sequences. The phylogenetic tree was constructed using the maximum likelihood method and the Jukes-Cantor model (bootstrap = 1,000) (Kumar et al., 2018).

### Average nucleotide identity (ANI) and digital DNA–DNA hybridization (dDDH)

The IPAC21 genome was compared with those of ten closely related *Paenibacillus* strains (listed in Table 1) using the JSpeciesWS database^4^ with the alignment algorithm blastn (ANIb) (Richter et al., 2016). The draft genomes were downloaded from NCBI^5^ and JGI^6^.

**Table 1:** Average nucleotide identity (ANI) and digital DNA–DNA hybridization (DDH) values between IPAC21 and closely related species of the genus *Paenibacillus.*

Query	Reference genomes	Access numbers	ANIb (%)	dDDH (%)
IPAC21	*Paenibacillus antarcticus* 20CM^T^ = CECT 5836	LVJI01000026.1	98.78 [90.76]*	91.2 [89.1–93]*
IPAC21	*Paenibacillus antarcticus* KACC 11469	NZ_CP043611.1	98.77 [90.75]	91.2 [89–93]
IPAC21	*Paenibacillus macquariensis* subsp. defensor JCM 14954	LVJG01000074.1	90.59 [69.02]	43.8 [41.3–46.4]
IPAC21	*Paenibacillus macquariensis* subsp. macquariensis DSM 2	LVJF01000065.1	90.52 [70.56]	43.8 [41.2–46.3]
IPAC21	*Paenibacillus macquariensis* ATCC 23464	FTNK01000072.1	90.51 [70.23]	43.7 [41.2–46.3]
IPAC21	*Paenibacillus glacialis* DSM 22343	LVJH01000032.1	86.55 [59.05]	33.10 [30.7–35.6]
IPAC21	*Paenibacillus crassostreae* LPB0068	LSFN01000012.1	76.22 [42.78]	21.20 [18.9–23.6]
IPAC21	*Paenibacillus xylanexedens* PAMC 22703	CP018620.1	68.57 [31.31]	24.80 [22.5–27.3]
IPAC21	*Paenibacillus tundrae* DSM 1314	708,558	68.50 [29.53]	19.9 [17.7–22.3]
IPAC21	*Paenibacillus borealis* DSM 13188	NZ_CP009285.1	68.19 [31.64]	22.60 [20.3–25]

* The numbers between parentheses after values of ANIb are the percentage of conserved aligned DNA between two genomes, and the numbers between brackets after dDDH values are the confidence intervals.

DNA digital hybridization (dDDH) was performed using the Genome-to-Genome Distance Calculator—GGDC 2.1 (Meier-Kolthoff et al., 2013) provided by Leibniz on the DSMZ Institute website^7^ with the recommended parameters and/or default settings.

### Comparative genomics

A comparative genome map was plotted through a BLASTN-based ring generated by BLAST ring image generator (BRIG) version 0.95 (Alikhan et al., 2011) to compare the draft genomes of six closely related *Paenibacillus* species with that of *P. antarcticus* strain IPAC21 used as a reference. A manual annotation of proteins was also performed using BLASTp and RAST, and the KEGG database^8^ was used to understand the possible metabolic pathways in which some proteins are embedded.

### Bioemulsifier production *in vitro*

#### Growth and production of the *Paenibacillus antarcticus* IPAC21 bioemulsifier in different culture media

Different culture media were compared for bioemulsifier production by strain IPAC21: TSB, LB (Luria Bertani), GB (glucose 10 g; peptone 10 g; meat extract 2 g; yeast extract 1 g; NaCl 5 g; H_2_O q.s. 1000 ml, pH 7.2), MM (NaCl 10 g; Na_2_HPO_4_ 9.44 g; (NH_4_)_2_SO_4_ 2 g; glucose 10 g; MgSO4.7H_2_O 0.2 g; H_2_O q.s. 1000 ml), MSS (NaCl 10 g; sucrose 10 g; Na_2_HPO_4_ 5 g; NH_4_NO_3 2_ g; KH_2_PO_4_ 2 g; MgSO_4_.7H_2_O 0.2 g; H_2_O q.s. 1000 ml). Flasks containing 50 ml of each medium were inoculated with approximately 5 × 10^8^ cells (OD600 = 0.6). The flasks were incubated at 28°C in a rotatory shaker (130 rpm) for 48 h, and 1 ml was centrifuged (13,000× *g*, 15 min). The cell-free supernatants were used for E24 determination.

### Determination of the emulsification index (E24)

The emulsification index (E24) was determined according to Iqbal et al. (1995) by the addition of 1 ml of n-hexadecane (or 1 ml diesel or 1 ml kerosene) to the same volume of cell-free supernatant in test tubes. The tubes were vortexed at high speed for 3 min. The stability of the emulsion was determined after 24 h at room temperature, and the emulsification index (E24) was calculated as the percentage of the height of the emulsified layer (mm) divided by the total height of the liquid column (mm). Tests were performed in triplicate. Statistical analyses were performed using PAST v4.02 software (Hammer et al., 2001) using one-way analysis of variance (ANOVA) followed by Tukey’s test.

### Drop collapse test

A volume of 10 μl from cell-free culture broth supernatant (strain IPAC21 grown at 28°C for 48 h) was dropped onto a crude oil-coated glass slide. Whenever the interfacial tension between the liquid and the hydrophobic surface was reduced, the drops spread or collapsed (Jain et al., 1991). The same volumes of sterile TSB and 10% SDS were used as negative and positive controls, respectively.

### Oil displacement test

A 90 × 15-mm Petri dish was filled with 40 ml of distilled water, and 20 μl of crude oil was added to form an apolar thin layer of hydrocarbons on the surface. Then, 10 μl of the cell-free culture supernatant (strain IPAC21 grown in TSB at 28°C for 48 h) was placed on the oil layer, and a positive result was considered when a clear halo was observed (Morikawa et al., 2000). The same volumes of sterile TSB and 10% SDS were used as negative and positive controls, respectively.

### Surface-activity determination

The cell-free culture supernatant of IPAC21 grown in TSB at 28°C for 48 h (1 ml) was analyzed on a Kruss DSA100 goniometer (Model: DE 3210) to determine the surface tension (ST) by the drop shape method, as described by Song and Springer (1996). To increase the accuracy of the surface tension measurements, an average of triplicates was conducted. All measurements were performed at room temperature.

### Determination of optimal physiological parameters for bioemulsifier production

Emulsification was analyzed during *P. antarcticus* IPAC21 growth in TSB (200 ml) at 28°C under agitation (130 rpm) for 96 h. Aliquots (5 ml) were collected after 24, 48, 72, and 96 h of cultivation to construct growth, absorbance (OD_600_), and emulsification curves. To determine the number of vegetative cells, 1 ml aliquots were serially diluted (up to 10^−5^), and each dilution was plated (100 μl) in TSB with the addition of 1.5% agar (TSA). The plates were incubated at 28°C for 48 h, and the colony forming units (CFU/ml) were calculated. The absorbance curve at OD_600_ was determined using 1 ml aliquots in a UV/VIS spectrophotometer (Mettler Toledo). The E24 assessments were determined following the same conditions previously described. All experiments were performed in triplicate, and the arithmetic means and standard errors were determined. Negative controls were performed using only TSB.

### Stability of the bioemulsifier produced by IPAC21 under different salinity, pH and temperature conditions

The effects of different chemical and physical parameters on the bioemulsifier produced by the IPAC21 strain were evaluated. Before E24 determination, the cell-free supernatants containing the bioemulsifier were submitted to different salinity, pH and temperature conditions. The salinity levels ranged from 3 to 12%, whereas the pH values varied from 2 to 12 (pH adjusted with either 6 N HCl or 2 N NaOH). The supernatant was maintained at −20°C, 5°C, 15°C for 15 min, 2 h and 24 h, 100°C for 20 min, and it was also autoclaved at 121°C for 15 min. The IPAC21 growth conditions as well as the E24 experiments followed the same conditions described above. Statistical analyses were performed using PAST v4.02 software as described previously.

## Results and discussion

### Phylogenetic analysis of the 16S rRNA encoding gene

**Figure 1:**
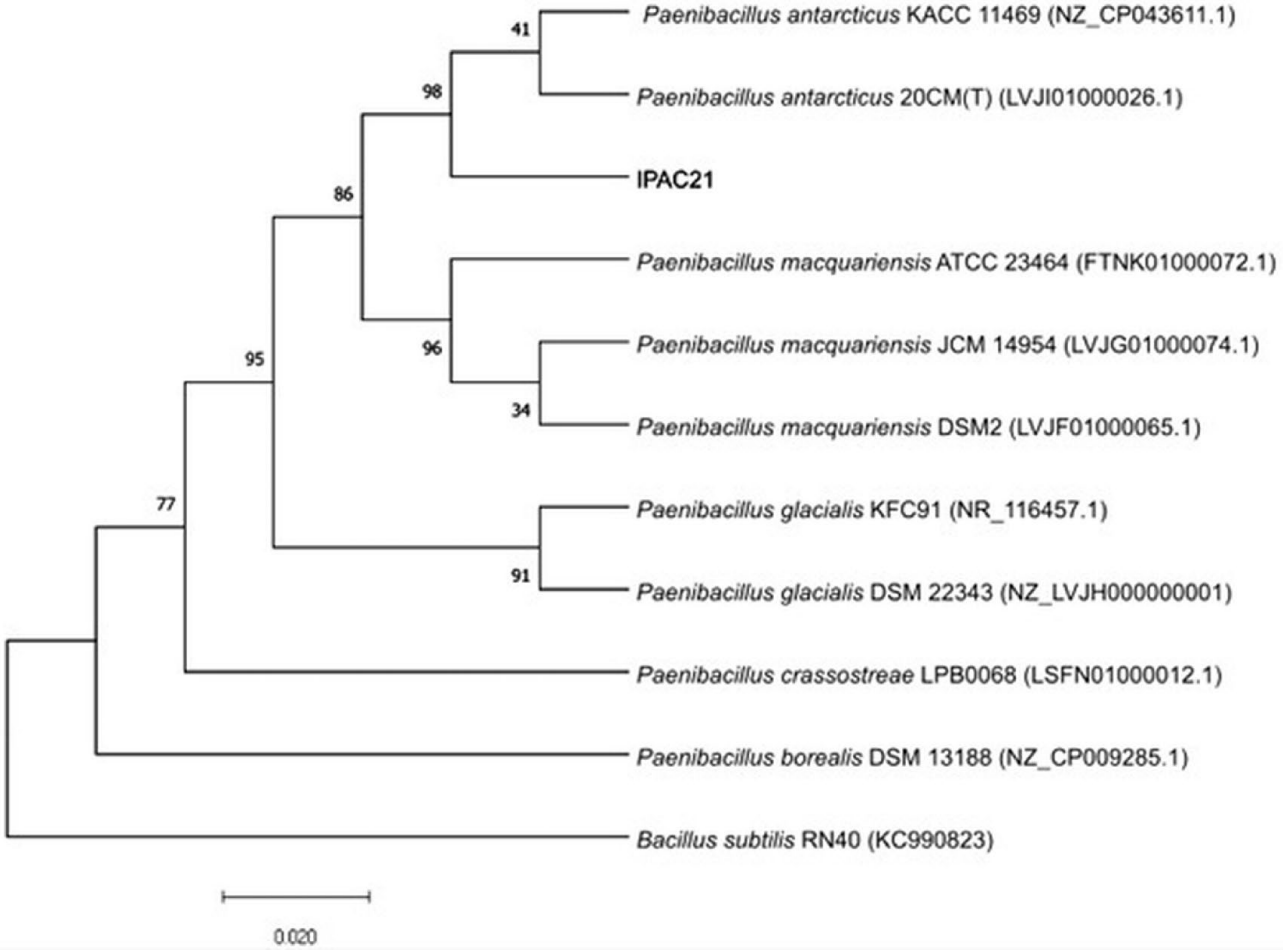
Alignment of the 16S rRNA-encoding gene of *Paenibacillus antarcticus* IPAC21 and related species. The phylogenetic tree was constructed using the maximum likelihood method and the Jukes–Cantor model. The GenBank accession number of each sequence is shown in parentheses. Bootstrap values are expressed as percentages of 1,000 replications and are shown at branch points. *Bacillus subtilis* RNA40 was used as an outgroup.

The results of BLAST sequence analyses of the 16S rRNA-encoding gene (1,510 bp) indicated that the strain, previously isolated from Antarctic soil and named IPAC21 (Vollú et al., 2014), is related to members of the genus *Paenibacillus* (Figure 1). Its closest relatives were *P. antarcticus* 20CM^T^ and *P. antarcticus* KACC 11469, with 99.93 and 99.85% gene sequence similarities, respectively.

### Genome annotation

The draft genome sequence of strain IPAC21 was determined in this study, and the whole genome shotgun project has been deposited at DDBJ/ENA/GenBank under the accession number BioProject PRJNA913778, BioSample ID SAMN32307255.

**Figure 2:**
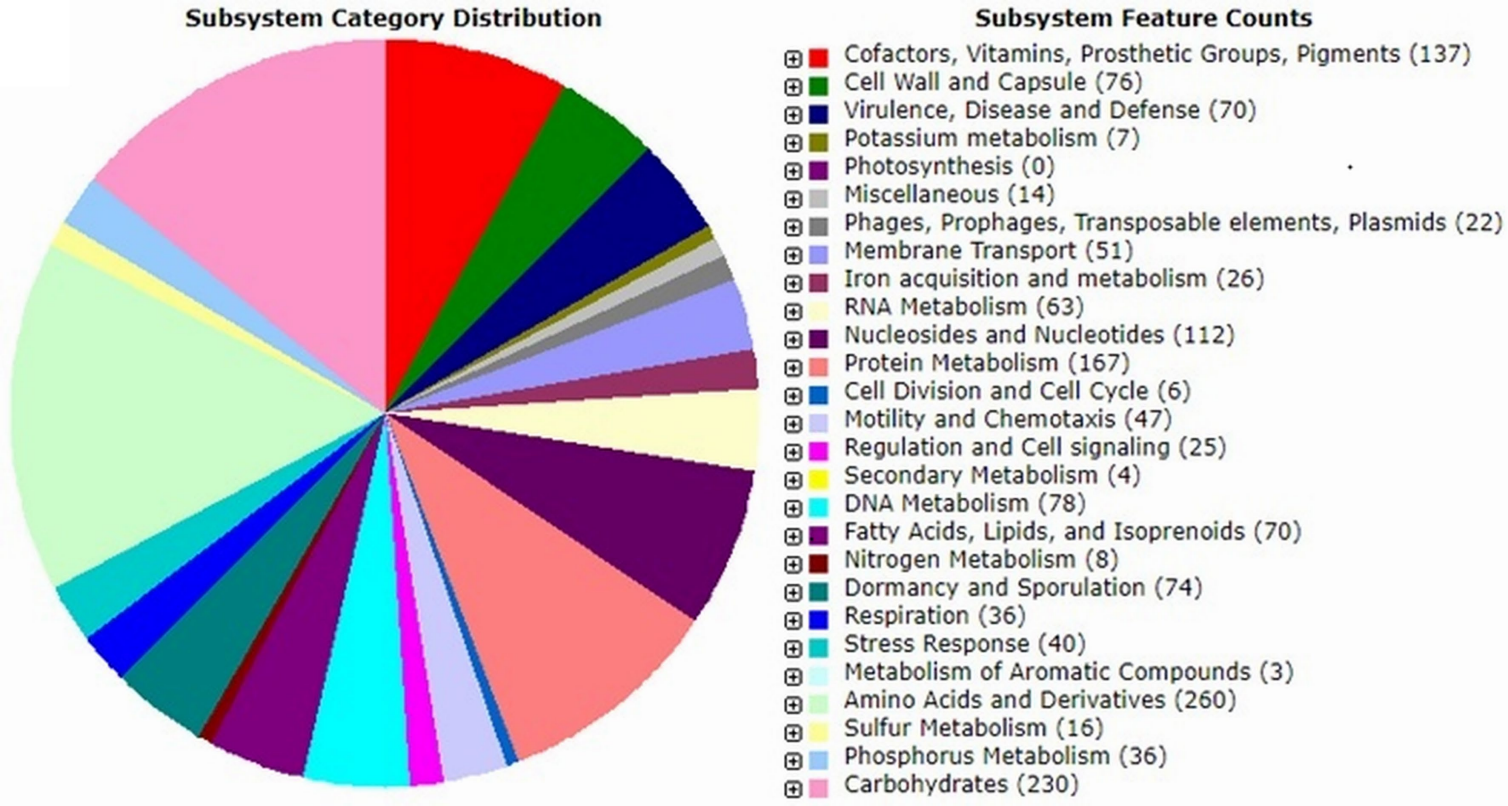
*Paenibacillus antarcticus* strain IPAC21 subsystem features based on the RAST annotation server.

The genome of strain IPAC21 revealed 5,505,124 bp with a G + C content of 40.5%, and a total of 5,240 coding DNA sequences (CDSs) were predicted. The identified CDSs were classified into subsystems, such as amino acids and derivates (260 CDSs), carbohydrates (230 CDSs), protein metabolism (167 CDSs), cofactors/vitamins/pigments (137 CDSs), stress response (40 CDSs), secondary metabolism (4 CDSs) and metabolism of aromatic compounds (3 CDSs) (Figure 2).

To explain the taxonomic relationship between the closely related *Paenibacillus* species and IPAC21 strain, the average nucleotide identity (ANI) and digital DNA–DNA hybridization (dDDH) values were determined between strain IPAC21 and the other ten type genomes of the members of the genus *Paenibacillus* (Table 1). The ANI values varied between 68.19 and 98.78%, where the accepted limit for species delimitation can be considered 95–96% (Richter and Rosselló-Móra, 2009). In addition, the results of DDH *in silico* were mostly below 70%, which is the cutoff value for species delimitation (Goris et al., 2007), with the exception of the value observed between the IPAC21 strain and either *P. antarcticus* 20CM^T^ or *P. antarcticus* KACC 11469, which was 91.2% (Table 1). Therefore, both the ANI and the DDH results suggest that the IPAC21 strain can be considered to belong to the species *P. antarcticus,* as previously suggested by Vollú et al. (2014).

### Genome features

#### 2,3-Butanediol fermentation pathway

The analysis of the *P. antarcticus* IPAC21 genome revealed the presence of the butanediol fermentation pathway. 2,3-Butanediol (2,3-BDO) is important as a liquid fuel additive, a softening and moistening agent, a solvent, a synthetic rubber precursor, an antifreeze

agent in different industries, including the petroleum industry (Celinska and Grajek, 2009; Dias et al., 2018). The *asl* gene encoding *R*-acetolactate synthase (EC 2.2.1.6), the *budA* gene encoding *R*-acetolactate decarboxylase (EC 4.1.1.5), and the *budB* gene encoding 2,3-butanediol dehydrogenase, R-alcohol forming, (R)- and (S)-acetoin-specific (EC 1.1.1.4) were identified in the IPAC21 genome. The presence of genes involved in butanediol fermentation suggests that IPAC21 is a facultative anaerobic strain similar to the strain *P. antarcticus* 20CM^T^ described by Montes et al. (2004). The *asl* and *butAB* genes have previously been demonstrated in the genome of *P. brasilensis* PB24 (Dias et al., 2018). However, while genes related to three butanediol isomers were detected in *P. brasilensis* PB24, only one isomer was observed in the IPAC21 and *P. polymyxa* PM 3605 genomes (Tinôco et al., 2021; this study).

### Adaptations to environmental stress conditions

Microorganisms in cold habitats need to face several adaptive challenges, such as ice formation that damages cell structures, low nutrient availability, high salinity, high UV irradiation, and oxidative stress (Margesin and Collins, 2019). To better understand the survival strategies of *P. antarcticus* IPAC21, genes associated with adaptation to environmental stress conditions, such as cold response, membrane fluidity mechanisms, DNA repair, oxidative and osmotic stress, chaperones and transport of compatible solutes, were highlighted in its genome.

Three cold shock-domain family proteins (Csps) were detected in the IPAC21 genome. Csps are a family of highly conserved small proteins that bind to single-stranded nucleic acids (Horn et al., 2007). These proteins are involved in metabolic processes of the stress response within the cell, such DNA replication, transcription, and translation (Casanueva et al., 2010; Chaudhary et al., 2022). The CspA family constitutes the main response to cold and is exclusive to psychrophilic bacteria (Leeson et al., 2000). CspA together with CspB acts as an RNA chaperone, destabilizing mRNA and improving translation efficiency at low temperatures (Aliyu et al., 2016; Garcia-Lopez et al., 2021). Other heat stress responses found in the IPAC21 genome are the DNAj and DNAk chaperones.

The repair system found in the IPAC21 genome involves genes that encode the SOS regulon proteins, consisting of three genes that encode the LexA repressor. These genes are a diverse family of bacterial transcription factors that repress genes in the cellular SOS response to DNA damage (Sánchez-Osuna et al., 2021). Furthermore, three genes involved in excision repair for UvrD were found. UvrD is a DNA helicase that participates in nucleotide excision repair and processes associated with replication (Newton et al., 2012).

DEAD-box RNA helicase and recAFON recombinases were also found in the IPAC21 genome. The *recA* gene product is a multifunctional enzyme that plays a role in homologous recombination, DNA repair and induction of the SOS response (Selbitschka et al., 1991). Microorganisms that are exposed to cold form unfavorable secondary mRNA structures, resulting in inhibition of translation. DEAD box RNA helicase is capable of reversing the secondary structure of the mRNA so that Csps can bind and prevent refolding before translation can take place (Phadtare and Severinov, 2010).

Low temperatures reduce membrane fluidity and permeability, and microorganisms respond by producing less saturated fatty acids to improve membrane fluidity (Chattopadhyay, 2006). In the genome of IPAC21, the enzyme fatty acid desaturase (EC 1.14.19.-) can be related to membrane fluidity that reduces fatty acid saturation and is upregulated at low temperatures (Rodrigues and Tiedje, 2008).

Genes related to oxidative stress were also identified in the IPAC21 strain, including four genes encoding catalases, seven coding for peroxidases, three for hydroperoxides, two for superoxide dismutase, three for thioredoxins, and ten for thioredoxin reductase. In cold environments, the solubility of oxygen increases, generating reactive oxygen species (ROS), resulting in DNA damage (Russo et al., 2010). Some mechanisms of adaptation to oxidative stress have already been described, such as enzymatic mechanisms that involve the use of enzymes superoxide dismutase (SOD), catalase (CAT) and/or glutathione peroxidase (GPx) (Barria et al., 2013). For example, the enzyme superoxide dismutase converts oxygen into hydrogen peroxide, which is less reactive and is an important antioxidant defense in cells exposed to oxygen. Similar to IPAC21, other bacterial strains found in Antarctica - *Serratia* sp. I1P (Monsalves et al., 2020), *Nesterenkonia* sp. AN (Aliyu et al., 2016) and *Bacillus weihenstephanensis* (den Besten et al., 2013) show this mechanism against ROS.

Two genes encoding proteins involved in adaptation to osmotic stress are also present in the IPAC21 genome, *opuAA* and *opuAB*, which are related to the ABC transport system of glycine/betaine and proline. Compatible solutes are compounds that protect and stabilize cellular components exposed to stress conditions without significantly interfering with their functions (Angelidis and Smith, 2003). Osmolyte transport proteins act as osmoprotectants and are involved in protection in environments with high osmolarity.

All the genetic features observed in the *P. antarcticus* IPAC21 genome suggest that this strain has developed survival strategies for its adaptation to environmental stress conditions found in Antarctic soils.

### Bioemulsifier production *in silico*

**Figure 3:**
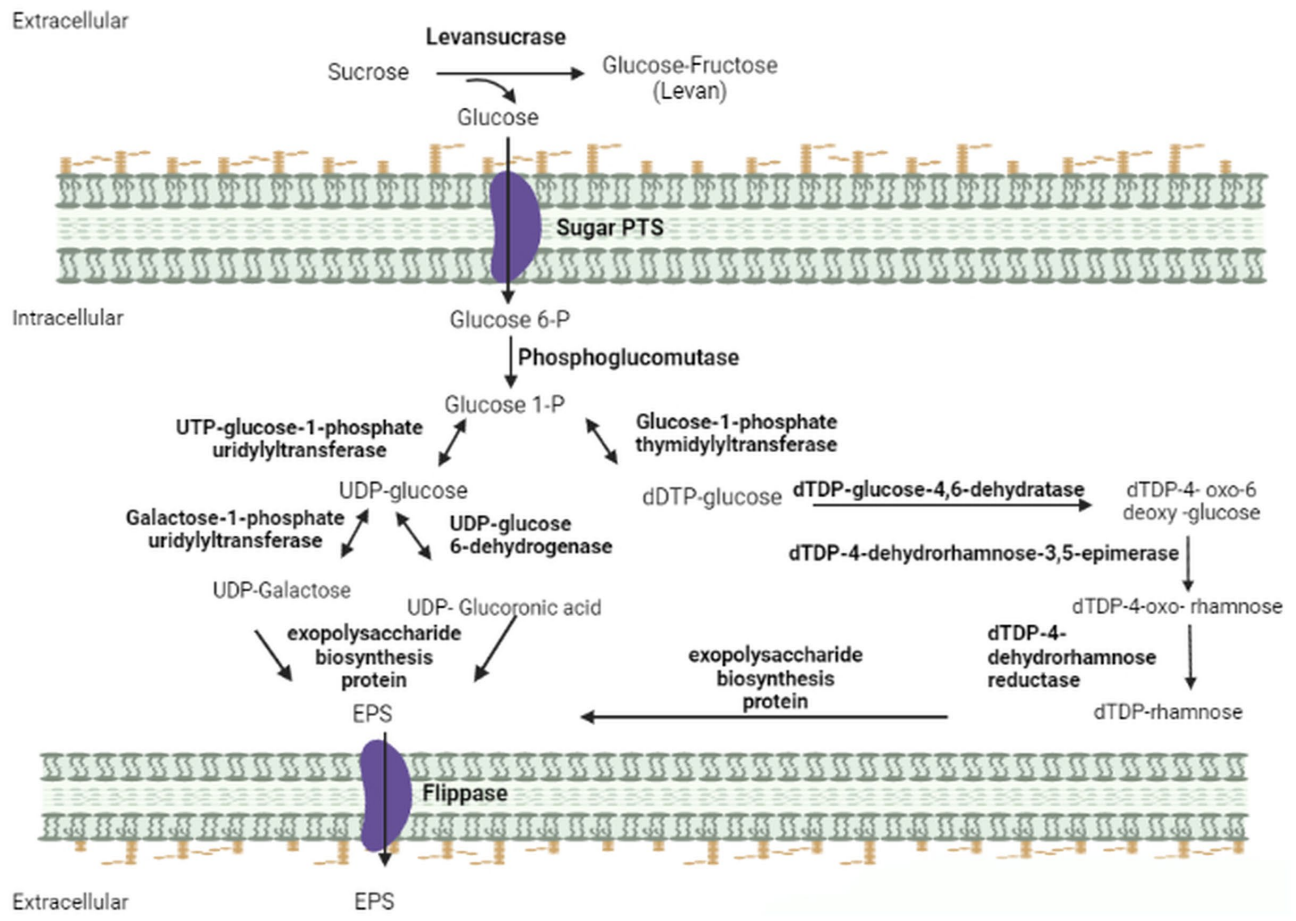
Representation of the metabolic pathway of EPS production found in the IPAC21 genome. The diagram demonstrates the enzymes found using KEGG and Metacyclic annotation programs. Levansucrase (EC 2.4.1.10); sugar PTS (EC 2.7.1.-); phosphoglucomutase (EC 5.4.2.2); UTP-glucose-1-phosphate uridylyltransferase (EC 2.7.7.9); glucose-1-phosphate thymidylyltransferase (EC 2.7.7.24); galactose-1-phosphate uridylyltransferase (EC 2.7.7.12); UDP-glucose-6-dehydrogenase (EC 1.1.1.22); dTDP-glucose-4,6-dehydratase (EC 4.2.1.46); dTDP-4-dehydrorhamnose-3,5-epimerase (EC 5.1.3.13); dTDP-4-dehydrorhamnose reductase (EC 5.1.3.- 1.1.1.-); exopolysaccharide biosynthesis protein; flippase (EC 7.6.2.1).

The analysis of the IPAC21 genome revealed the presence of the gene that encodes the extracellular enzyme levansucrase (EC 2.4.1.10) responsible for the synthesis of levan (Figure 3). Levan is a naturally occurring fructan, the homopolymer of fructose, found in many plants and microbial products, and it has many applications in different industrial fields, such as foods and pharmaceuticals (Mummaleti et al., 2022). Its main chain is composed of repeating five-member fructofuranosyl rings connected by β-(2 → 6) links. The main chain is branched through the β-(2 → 1) linkage of the fructofuranosyl rings (Mendonça et al., 2021). The production of levan starts in the extracellular medium through the conversion of sucrose to fructose/glucose by the enzyme SacB, which synthesizes fructose polymers through a transfructosylation reaction using sucrose as a donor fructose/glucose (Xu et al., 2021). The production of levan has already been described in other *Paenibacillus* species, such as *P. polymyxa* DSM 365 (Okonkwo et al., 2020), *Paenibacillus* sp. #210 (Mendonça et al., 2021) and *P. bovis* BD3526 (Xu et al., 2016).

The *sacB* gene encoding levansucrase was found in the IPAC21 genome with 97% similarity with that of *P. antarcticus* 20CM^T^. The genes coding for phosphoglucomutase (EC 5.4.2.2), responsible for the first reaction of the pathway, uridyltransferase (EC 2.7.7.23), UDP-glucose 6-dehydrogenase (EC 1.1.1.22), glucose-1-phosphate thymidyltransferase (EC 2.7.7.24), dTDP-glucose 4,6-dehydratase (EC 4.2.1.46), and three genes for exopolysaccharide biosynthesis proteins (EC 2.4.1.-) were also observed (Figure 3).

In addition, transmembrane transporters responsible for the acquisition of sugar compounds were detected in the genome of the IPAC21 strain. The presence of 483 transporters, of which 366 showed similarities with transporters ABC (ATP-binding system cassette) and 11 PTS (phosphoenolpyruvate-dependent sugar phosphotransferase system), was observed. Both transport systems act in the transport of several types of sugars, such as D-glucose (EC 2.7.1.-), D-fructose (EC 2.7.1.-), D-galactose (EC 7.5.2.11), maltose (EC 7.5.2.1), lactose (EC 7.5.2.2) and arabinose (EC 7.5.2.7). Furthermore, twenty-three MFS-type transporters, seven drug/metabolite transporters, and four HrtAB transporters were also identified in the IPAC21 genome.

### Comparative genomics

**Figure 4:**
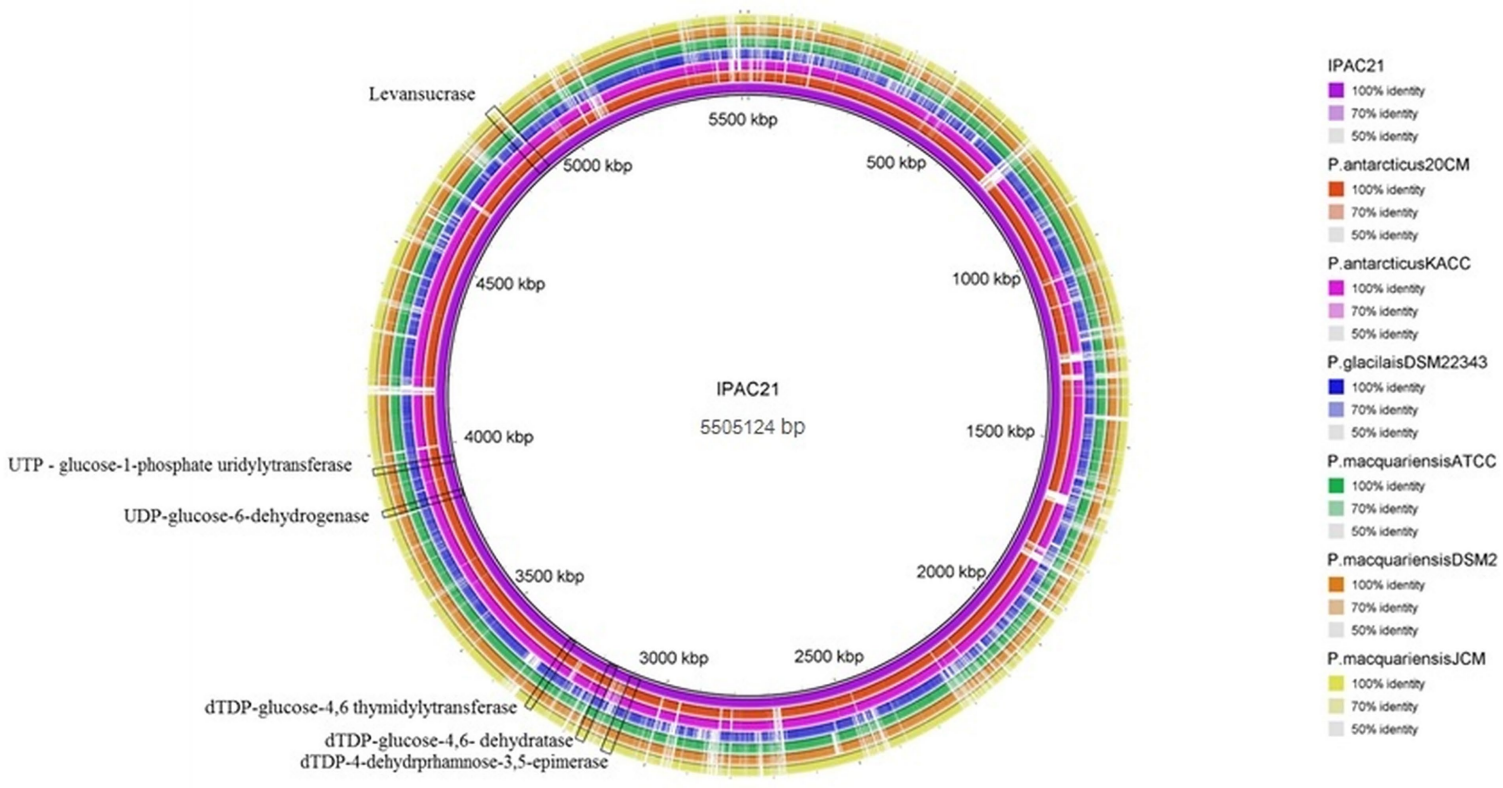
Circular diagram illustrating the nucleotide similarity between *P. antarcticus* IPAC21 (in purple, inside the circle) and other *Paenibacillus* genomes represented by concentric rings.

The similarity between the DNA regions involved in bioemulsifier production between IPAC21 and related species is highlighted in the comparative genome map (Figure 4). The similarity between the *sacB* gene that produces a levansucrase enzyme in the genome of IPAC21, of *P. antarcticus* 20CM^T^, and *P. antarcticus* KACC 11469 presents a value of 87.7 and 87.5% identity, respectively. *Paenibacillus* species such as *P. glacialis* DSM 22343, *P. macquariensis* ATCC 23464, *P. macquariensis* DSM 2, and *P. macquariensis* JCM 14954 showed an identity of less than 55% when compared to IPAC21.

### Bioemulsifier production *in vitro*

#### Growth and production of the *Paenibacillus antarcticus* IPAC21 bioemulsifier in different culture media

Strain IPAC21 was able to grow in four media tested (TSB, LB, GB and MM). The highest number of cells and E24 were obtained in TSB. The emulsification activity with n-hexadecane was directly proportional to the growth of *P. antarcticus* IPAC21 and reached the highest value (63.7% ±1.7) at the end of the log phase (48 h), corresponding to approximately 1.5 × 10^9^ CFU/ml and an absorbance of 1.7.

The cell-free supernatant obtained after IPAC21 growth in TSB was submitted to drop collapse, oil displacement tests and superficial tension tests. Negative results were obtained in all tests. No capacity to reduce surface tension (68.69 mN/m) was observed. These results are expected for bioemulsifiers, which are molecules that emulsify two immiscible liquids, such as hydrocarbons or other types of hydrophobic substrates, even at low concentrations and are less effective in reducing surface tension (Uzoigwe et al., 2015). Bioemulsifiers have already been described in other mesophilic *Paenibacillus* species, such as *P. polymyxa* (Gudina et al., 2015), *P. dendritiformis* CN5 (Bezza and Chirwa, 2017), and *Paenibacillus* sp. 210 (Mendonça et al., 2021). In cold environments, such as Antarctica, other bacterial species are also producers of bioemulsifiers and/or biosurfactants and are considered reservoirs of new biotechnological molecules (Perfumo et al., 2018; Schultz and Rosado, 2020).

### Emulsification of hydrocarbons by *Paenibacillus antarcticus* IPAC21

**Figure 5:**
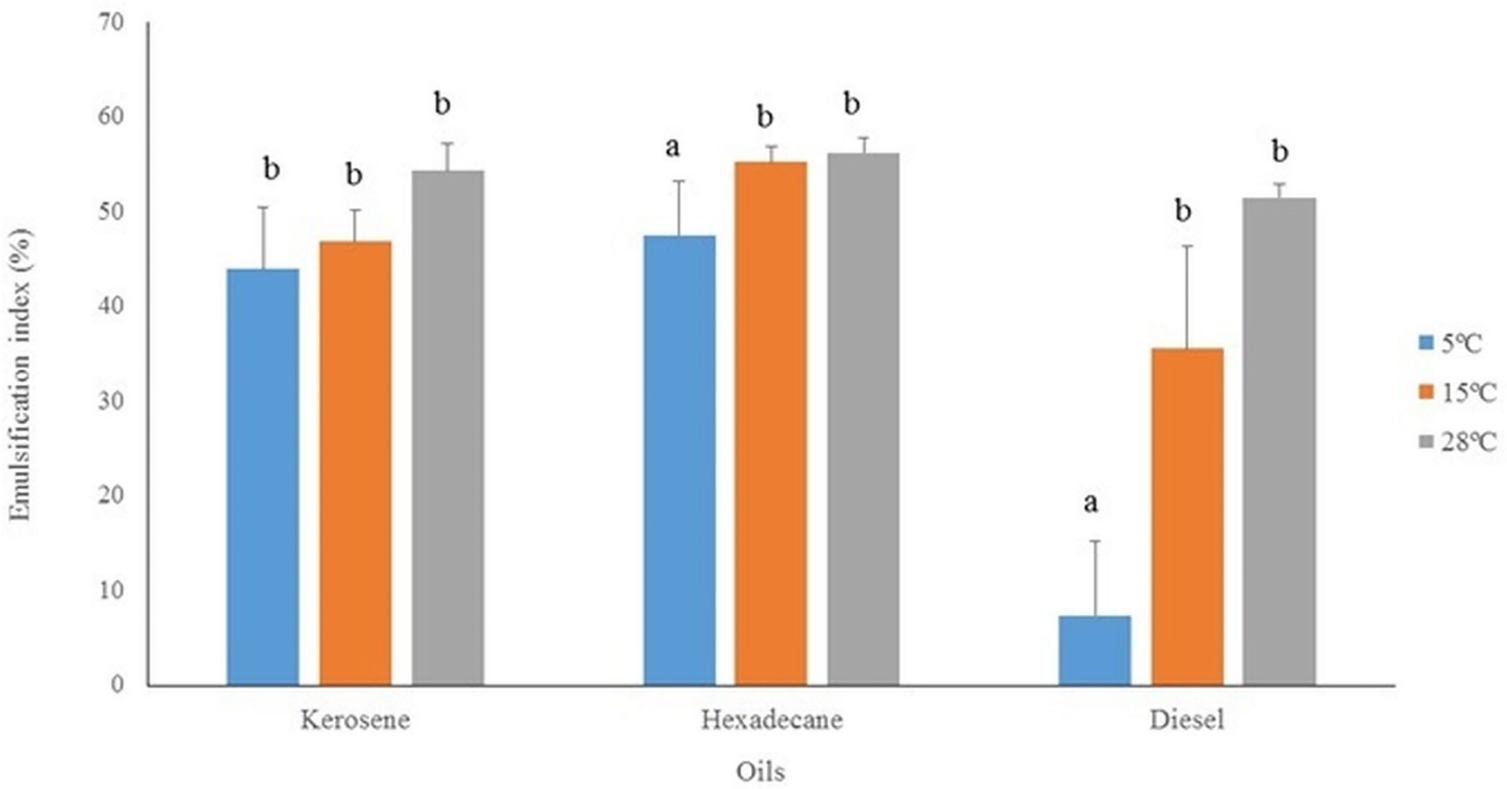
Emulsifying indexes (E24) of *P. antarcticus* IPAC21 with different oils (kerosene, n-hexadecane, diesel) after IPAC growth in TSB at 5°C, 15°C, and 28°C. Different letters indicate statistically significant differences (Tukey, *p* < 0.05).

The bioemulsifier produced by *P. antarcticus* IPAC21 was evaluated for its potential application in the oil industry. E24 experiments were conducted with kerosene, hexadecane and diesel at three different temperatures (5°C, 15°C, and 28°C). IPAC21 showed emulsification in the three types of oils and at the three temperatures tested, with the highest values at 28°C and the lowest at 5°C (Figure 5). The highest emulsification value was for n-hexadecane at 28°C (E24 = 56.2% ±1.5), followed by the temperature of 15°C (E24 = 55.2% ±1.6), and at 5°C (E24 = 47.5% ±5.6). The emulsions maintained their stability for at least 30 days. No significant differences (*p* > 0.05) were observed in the E24 values using kerosene at the three temperatures tested. However, using hexadecane and diesel, significant differences (*p* < 0.05) were observed at a temperature of 5°C when compared to 15°C and 28°C (Figure 5).

### Stability of the bioemulsifier produced by IPAC21 under different salinity, pH and temperature conditions

**Figure 6:**
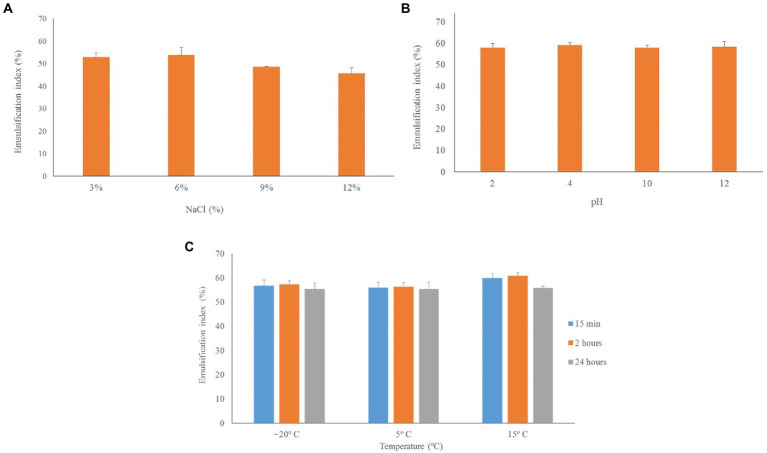
Stability of the bioemulsifier produced by IPAC21 under different salinity, pH and temperature conditions. **(A)** NaCl concentrations of 3, 6, 9 and 12%. **(B)** pH of 2, 4, 10, 12. **(C)** Temperatures of −20°C, 5°C and 15°C.

The effect of salinity, pH, and temperature was evaluated from cell-free supernatant containing the bioemulsifier produced by IPAC21 in TSB at 28°C for 48 h, as shown in Figure 6. The E24 values for the addition of NaCl ranged from 45.6 ± 2.2 to 53.8 ± 1.2%, with the highest value for the salt concentration in the supernatant being 6%, whereas the lowest value for the salt concentration was 12%. Regarding the pH variation, the E24 values were very close, with the highest value of E24 = 59.1 ± 0.9% observed at pH 4 and the lowest value of E24 = 57.9 ± 0.5% at pH 10. At low temperatures, the lowest E24 value (55.6 ± 2.2%) was observed at 5°C for 15 min, and the highest value (60.9 ± 1.3%) was observed at 15°C for 2 h. No statistically significant differences in the abovementioned stability tests were observed (Tukey’s test, *p* > 0.05) (Figure 6). Regarding the heat treatment, the values of the emulsification index were negligible: E24 = 6.8 ± 3.4% for the heat treatment at 100°C for 20 min and E24 = 8 ± 3.5% for the heat treatment by autoclaving.

Other *Paenibacillus* strains showed similar responses as those observed in IPAC21 to salt and pH variations. The E24 values of the bioemulsifier produced by *Paenibacillus* sp.#510 were almost constant (56.2–59.7%) over the entire pH range tested (Gudina et al., 2015). Likewise, only small variations were observed in the E24% with the addition of NaCl and over a wide range of pH values in *P*. *alvei* ARN63 (Amirabadi et al., 2013). However, the sensitivity to high temperature observed in the IPAC21 bioemulsifier was not shared by *Paenibacillus* sp. # 510 and *P. alvei* ARN63. Heat treatment did not reduce the emulsion formation capacity of the bioemulsifiers produced by these strains (Amirabadi et al., 2013; Gudina et al., 2015). This variation in thermal stability can be explained mainly by the differences in the structure, density and molecular weight of the bioemulsifiers and in fermentation parameters, including the medium and the microorganism used for their production (Mummaleti et al., 2022).

## Conclusion

In this study, the genome of *P. antarcticus* IPAC21 was presented, showing the presence of genes associated with adaptation to environmental stress conditions such as cold response, membrane fluidity mechanisms, DNA repair, oxidative and osmotic stress, chaperones and transport of compatible solutes. Genes for the biosynthesis of the bioemulsifier levan are described and compared to those of closely related *Paenibacillus* species. To better characterize the bioemulsifier produced by IPAC21, emulsification experiments were performed, and emulsification values using kerosene and hexadecane at three different temperatures (5°C, 15°C, and 28°C) were close to 50% or higher. The bioemulsifier was stable at different NaCl concentrations, low temperatures and pH values but not at high temperatures. All the data obtained contribute to a better knowledge of this psychrotolerant strain isolated from Antarctic soil, showing potential biotechnological applications in the petroleum industry. Given the great stability of the bioemulsifier produced by *P. antarcticus* IPAC21, even in a wide range of temperature, pH, and NaCl, we see as extremely relevant the future study concerning its effectiveness in bioremediating different environments (including cold marine environments) contaminated with pollutants derived from extracting and/or transporting petroleum.

## Data availability statement

The datasets presented in this study can be found in online repositories. The names of the repository/repositories and accession number(s) can be found in the article/supplementary material.

## Author contributions

EL, DJ, and LS conceived and designed the study. EL conducted the experiments. LP, AR, and FM contributed to the genomic data analyses. EL, LP, and LS wrote the first draft of the manuscript. All authors revised the manuscript, provided comments, and approved the final version of the manuscript.

## Funding

This study was supported by grants from Conselho Nacional de Desenvolvimento Científico e Tecnológico (CNPq), Coordenação de Aperfeiçoamento de Pessoal de Nível Superior (CAPES – financial code 001) and Fundação de Amparo à Pesquisa do Estado do Rio de Janeiro (FAPERJ). AR was supported by KAUST Baseline Grant (BAS/1/1096-01-01).

## Conflict of interest

The authors declare that the research was conducted in the absence of any commercial or financial relationships that could be construed as a potential conflict of interest.

## Publisher’s note

All claims expressed in this article are solely those of the authors and do not necessarily represent those of their affiliated organizations, or those of the publisher, the editors and the reviewers. Any product that may be evaluated in this article, or claim that may be made by its manufacturer, is not guaranteed or endorsed by the publisher.

## Footnotes

^1^ http://lpsn.dsmz.de

^2^ www.genome.jp/kegg

^3^ https://metacyc.org/

^4^ http://jspecies.ribohost.com/jspeciesws/

^5^ www.ncbi.nlm.nih.gov/refseq

^6^ https://genome.jgi.doe.gov/portal/

^7^ http://ggdc.dsmz.de/distcalc2.php

^8^ www.genome.jp/kegg
